# Reserved Force

**Published:** 1881-11

**Authors:** 


### RESERVED FORCE.

IT is not wise to work constantly up to the rate of which we are capable. If the engineer of a railroad were to keep the speed of his train up to the highest rate he could obtain with his engine it would soon be used up. If a horse is driven at the top of his speed for any length of time, he is ruined. It is well enough to try the power occasionally of a horse or engine by putting on all the motion it will bear, but not continuously. All machinists construct their machines so there shall be a reserve force. If the power required is a four-horse, then they make a six-horse power. In this case it works easily and lasts long. A man who has strength enough to do twelve honest hours of labor in the twenty-four, and no more, should do but nine or ten hours' work. The reserve power keeps the body in repair. It rounds out the frame to full proportions. It keeps the mind cheerful, hopeful, happy. The person with no reserve force is always incapable of taking on any more responsibility than he already has. A little exertion puts him out of breath. He cannot increase his work for an hour without danger of an explosion. Such are generally pale, bloodless, nervous, irritable, despondent, gloomy. We all pity them. The great source of power in the individual is the blood. It runs the machinery of life, and upon it depends our health and strength. A mill on the stream where water is scanty can be worked but a portion of the time. So a man with but little good blood can do but little work. The reserve power must be stored up in his fluid. It is an old saying among stock-raisers that "blood tells." It is equally true that blood tells in the sense in which we use the word. If it is only good blood, then the more of it the better. When the reserve power of an individual runs low, it is an indication that a change is necessary, and that it is best to stop expending and go to accumulating, just as the miller does when the water gets low in the pond. Such a course would save many a person from physical bankruptcy.

---

THE man who easily and quickly forgets a good turn is just the man to avoid doing one if he possibly can.

HOME love is the best love. The love that you are born to is the sweetest you will ever have on earth. You, who are so anxious to escape from the home-nest, pause a moment and remember this is so.

